# The impact of symptom distress on health-related quality of life in liver cancer patients receiving arterial chemoembolization: the mediating role of hope

**DOI:** 10.1186/s12876-022-02529-x

**Published:** 2022-11-15

**Authors:** Nan-Yan Chen, Kang-Hua Chen, Yi-Wen Wang, Hsiu-Hsin Tsai, Wei-Chen Lee, Li-Chueh Weng

**Affiliations:** 1 Department of Nursing, Linkuo Medical Centre, Chang Gung Medical Foundation, 333 Taoyuan, Taiwan; 2 School of Nursing, College of Medicine, Chang Gung University, 333 Taoyuan, Taiwan; 3 Department of General Surgery, Linkuo Medical Centre, Chang Gung Medical Foundation, 333 Taoyuan, Taiwan

**Keywords:** Hepatocellular carcinoma, Liver cancer, Arterial embolization, Hope, Quality of life

## Abstract

**Background:**

Trans-hepatic arterial chemoembolization (TACE) is a treatment option for liver cancer patients. It can prolong patients’ survival but can also cause symptom distress. Symptom distress (SDs) can directly impact quality of life (QOL) and may indirectly influence QOL by lessening hope. In this study, we wanted to explore the mediating effect of hope on the relationship between SDs and QOL among patients with liver cancer receiving TACE.

**Methods:**

A cross-sectional study was conducted from December 20, 2017, to August 6, 2018, at a gastroenterology ward of a medical center. The participants were 92 liver cancer patients (69.6% male, mean age 67.8) who were admitted for TACE treatment. Information on SDs, hope, and QOL was collected by questionnaire on discharge day. Hayes’ PROCESS model was used to test the mediating effect of hope on the relationship between SDs and QOL.

**Results:**

The mean score and standard deviation (SD) of SDs, hope, and QOL were 32.08 (SD = 6.22), 27.09 (SD = 3.51), and 55.16 (SD = 17.33), respectively. SDs negatively impacts quality of life. The total effect of SDs on QOL was − 1.41 (95% confidence interval [CI]: − 1.96, − 0.86). The indirect effect via the mediation of hope was − 0.95 (95% CI: − 1.7, − 0.45). Hope partially mediated the effect of SDs on QOL.

**Conclusion:**

SDs after TACE is vital; it directly reduces a patient’s overall QOL and can indirectly hinder it by reducing the patient’s hope. In addition to symptom management, interventions that help patients maintain their hope are key to improving QOL among patients receiving TACE.

## Background

Hepatocellular carcinoma (liver cancer) is prevalent globally and has a high mortality rate, especially in Asia [1–3]. Tumor characteristics, extrahepatic metastasis, vascular involvement, response to treatment, and the patient’s age, physical function, and mental function status are influencing factors related to the progress of liver cancer [1, 4]. In addition to mortality and survival, health-related quality of life (QOL), which includes psychological, physical, and social aspects, is a good care indicator for patients with cancer [5, 6]. Maintaining a proper QOL is also an expectation of cancer patients receiving advanced treatment [2, 7–9]. Nearly 80% of liver cancer patients are diagnosed as severe cases and are unable to undergo surgical resection [10]. Trans-hepatic arterial chemoembolization (TACE) is the primary therapy of choice for patients with liver cancer who cannot undergo surgical resection; it can also be used as an adjuvant therapy for liver cancer patients after surgery [11, 12]. TACE has been reported as an effective procedure for local and advanced liver cancer. The median survival time by cancer stage ranged from 15.8 to 49 months among patients receiving TACE [4]. TACE alone or combined with other therapy had better overall survival and time to progression than other treatments of liver cancer [1]. However, studies had shown that QOL was not good in patients receiving TACE [13, 14]. Poor QOL can further decrease the willingness of patients to complete the treatment course [15]. Therefore, factors that can compromise QOL in patients receiving TACE need to be investigated and managed continually.

TACE is a treatment that provides chemotherapy medication through the hepatic artery, inducing a potent attack at the tumor cell directly. TACE is similar to systemic chemotherapy, which may cause systemic side effects and uncomfortable symptoms such as pain, nausea, vomiting, fever, fatigue, poor appetite, abdominal fullness, sadness, anxiety, and sleep problems [8, 13, 16]. Symptom distress (SDs) is a subjective gauge of one’s discomfort from symptoms. SDs could seriously impair QOL after TACE [13, 17]. The effect of SDs on QOL needs further investigation in order to develop a tailored intervention to reduce the impact of SDs.

Hope is a mental process that can help patients with cancer cope with the physical and psychological impacts of the disease as well as maintain their mental well-being and QOL [7, 18–23]. A systematic review of 33 articles indicated that hope was positively correlated with QOL in cancer patients. Patients who had high hope reported higher life satisfaction and better health conditions as compared to those with low hope. The review also noted that symptom burden and mental fatigue were negatively correlated with hope [21]. In addition, persistent pain has a negative effect on hope [23]. However, the relationships among hope, SDs and QOL have not been well investigated.

The aim of this study was to examine the effect of SDs on QOL while considering the mediating effect of hope. Our hypotheses were that SDs directly influenced QOL and that SDs also influenced QOL indirectly through its effect on hope.

## Methods

### Study design and participants

A cross-sectional, correlational design was used. Data were collected from questionnaires, scales, and medical records. The study was conducted from December 20, 2017, to August 6, 2018, at a gastroenterology ward of a medical center in Northern Taiwan. A convenience sampling method was used to recruit the study sample. The inclusion criteria were patients who (1) had primary liver cancer, (2) knew about their diagnoses, (3) were only receiving TACE during their admissions, (4) could communicate in Mandarin, and (5) were aged 20 years or older. The exclusion criterion was the occurrence after TACE of severe complications such as bleeding.

The sample size was estimated using G*Power version 3.1 [24]; an effect size of 0.15, (medium effect size) [25], a significance level of 0.05, a power of 0.8, and regression-based statistical analysis were chosen. Ninety-eight participants were required.

### Measurements

#### Demographic and disease-related information

The demographic data included age, sex, education level, marital status, employment, monthly income, and religion. The disease-related characteristics included Barcelona Clinic Liver Cancer (BCLC) stage, first-time TACE treatment, duration of liver cancer diagnosis (months), number of TACE treatments received, and length of hospital stay (days). A questionnaire about basic demographic information and disease-related characteristics was developed by the authors. Five clinical experts (two physicians and three senior clinical nurses) were invited to evaluate its content validity. The content validity index ranged from 0.8 to 1 for single items, and the content validity index was 0.98 for the total questionnaire.

#### Symptom distress

The Symptom Distress Scale-Chinese Modified Form (SDS-CMF) was used to measure SDs after TACE [26, 27]. Permission to use this scale was obtained. There were 25 symptoms listed that were common in patients with liver cancer who received cancer-related treatment. Each symptom was scored on a Likert scale ranging from 1 (*never bothered*) to 5 (*severely bothered*). Possible scores ranged from 25 to 125, with a higher score indicating a higher level of SDs. The internal consistency reliability (Cronbach’s α) of the SDS-CMF in this study was 0.66.

#### Hope

The Chinese version of the Herth Hope Index (HHI) was used to measure hope [28]. Permission to use this scale was obtained. The HHI has been used worldwide [20] in studies of individuals in both hospital and community settings who experienced varying health conditions. The Chinese version comprises 10 items. Each item was scored on a Likert scale ranging from 1 (*strongly disagree*) to 4 (*strongly agree*). Possible scores ranged from 10 to 40, with a higher score indicating a higher level of hope. The internal consistency reliability (Cronbach’s α) of the HHI in this study was 0.89. We used exploratory factor analysis, principal component followed by varimax rotation to examine the validity of this scale in this study [29]. A two-factor structure for the 10-item HHI scale was identified. The percentage of variance explained was 63.75%, which indicated an acceptable validity.

### QOL

The Chinese version of the Functional Assessment of Cancer Therapy–General (FACT-G) was used to measure the QOL of patients with liver cancer after TACE; it was developed from the Functional Assessment of Chronic Illness Therapy [17, 30]. Permission to use this scale was obtained. The scale contains 27 items and measures physical (7 items), social/family (7 items), emotional (6 items), and functional (7 items) well-being on a 5-point Likert scale ranging from 0 (not at all) to 4 (very much). The possible FACT-G scores ranged from 0 to 108, with a higher score indicating a higher QOL. The Cronbach’s α of the FACT-G total scale and the physical, social/family, emotional, and functional subscales in this study were 0.95, 0.89, 0.84, 0.95, and 0.93, respectively.

### Data collection

After the study hospital’s institutional review board approved the study, the purpose and procedures were explained thoroughly to the participants and written consent was obtained from them. A set of questionnaires was used to collect the data. Basic demographic and disease-related data were collected upon admission. Questionnaires about SDs, hope, and QOL were collected on the discharge day.

### Data analysis

Data were analyzed using SPSS version 22.0 (IBM Corporation, Armonk, NY, USA). Mean, standard deviation (SD), count, and percentage were used to describe the distribution of the study variables. Pearson’s correlations, independent t-tests, and a one-way analysis of variance (ANOVA) were used to analyze the relationships among basic demographic variables, disease-related variables, and QOL. Significant demographic and disease-related variables were controlled for in the mediation model, which was analyzed using the mediation package PROCESS (version 3.5 by Professor Andrew F. Hayes for SPSS [31]). Statistical significance was set at p < .05. The mediating effect (indirect effect) was tested with bias-corrected bootstrapping (N = 5,000) and 95% confidence intervals (CIs) for the indices. When a 95% bootstrapped CI did not include zero, the indirect effect was statistically significant [31].

## Results

### Participants’ demographic characteristics

During the data collection period, there were 110 patients admitted for TACE treatment. Of these, 106 met the inclusion criteria, and 92 patients agreed to participate and completed the data collection (7 showed no interest and 7 worried about feeling uncomfortable after TACE). The proposed sample size was not achieved. Therefore, a post hoc Power analysis was taken, and results showed a power of 1. The participants’ characteristics were presented in Table 1.

**Table 1:** Participants’ characteristics (N = 92)

Variables	N	%	Mean	SD	Range
Age			67.8	8.74	48–85
Sex					
Female	28	30.4			
Male	64	69.6			
Education level					
Primary school	45	48.9			
Junior high school	22	23.9			
High school	25	27.2			
Marital status					
Unmarried/widow	9	9.8			
Married	83	90.2			
Employment					
Unemployed	3	3.3			
Employed	20	21.7			
Retired	69	75.0			
Monthly income (US$)					
0	66	71.6			
(0,1000]	11	12.0			
≥1001	15	16.4			
Practice a religion					
No	15	16.3			
Yes	77	83.7			
Taoist	49	63.6			
Buddhist	18	23.4			
Christian	4	5.2			
Catholic	1	1.3			
Kuan Taoist	5	6.5			
BCLC stage					
A	44	47.8			
B	37	40.2			
C	11	12			
Liver function (Child-Pugh)					
A	76	82.6			
B and C	16	17.4			
First-time TACE					
No	70	76.1			
Yes	22	23.9			
Duration of diagnosis (months)			27.42	31.03	0-148
Number of TACE treatments received			2.82	3.36	0–17
Length of hospital stay (days)			7.04	2.78	3–15
SDs			32.08	6.22	25–49
Hope			27.09	3.51	19–35
Overall QOL			55.16	17.33	13–86
Physical health domain			17.43	6.21	2–27
Social/family domain			11.25	4.08	0–25
Emotional domain			15.11	5.58	2–24
Functional domain			11.37	5.46	1–25

Note: BCLC = Barcelona Clinic Liver Cancer; QOL = quality of life; SD = standard deviation; SDs = symptom distress

### SDs, hope, and QOL

The mean scores of SDs, hope, and QOL on discharge day were 32.08 (SD = 6.22), 27.09 (SD = 3.51), and 55.16 (SD = 17.33), respectively. The mean scores of each domain of QOL were as follows: 17.43 for physical (SD = 6.21), 11.25 for social/family (SD = 4.08), 15.11 for emotional (SD = 5.58), and 11.37 for functional (SD = 5.44) (Table 1). The score of overall QOL was used as the dependent variable in Pearson’s correlations, independent t-tests, the one-way ANOVA, and mediation model analysis.

### Factors associated with QOL

Employed patients (mean = 62.65, SD = 15.91) had a higher overall QOL than did unemployed and retired patients (mean = 53.08, SD = 17.23, t = − 2.23, p = .028). The overall QOL differed among the BCLC stages (F = 3.42, p = .037). Scheffe’s post-hoc analysis indicated that patients with BCLC stage A (mean = 58.29; SD = 15.44) had higher QOL than patients with BCLC stage C (mean = 43.82; SD = 21.03). There were no statistically significant QOL differences based on sex, education level, marital status, monthly income, religion, or first-time TACE (Table 2).

**Table 2:** QOL comparisons among variables

Variables	Category	Mean	SD	t/F	p
Sex	Female	54.18	16.02	− 0.36	0.721
	Male	55.59	17.98		
Education level	Primary school	52.64	17.13	2.12	0.126
	Junior high school	53.50	18.08		
	High school	61.16	16.22		
Marital status	Unmarried/widow	49	20.59	− 1.13	0.264
	Married	55.83	16.95		
Employment	Unemployed ^a^	53.08	17.23	− 2.23	0.028*
	Employed	62.65	15.91		
Monthly income (US$)	0	52.82	17.07	2.24	0.112
	(0,1000]	59.91	17.88		
	≥1001	62.62	16.63		
Practice a religion	No	56.33	19.13	0.28	0.777
	Yes	54.94	17.08		
BCLC stage	A	58.29	15.44	3.42	0.037*
	B	54.46	17.25	(A > C)	
	C	43.82	21.03		
Liver function	A	54.49	17.84	− 0.81	0.420
	B and C	58.38	14.71		
First-time TACE	No	55.24	16.88	0.08	0.938
	Yes	54.91	19.09		

Note: ^a^ Unemployed = unemployed + retired; BCLC = Barcelona Clinic Liver Cancer; TACE = Trans-hepatic arterial chemoembolization; QOL = quality of life; SD = standard deviation; * p < .05

QOL was positively correlated with hope (r = .78, p < .001) but negatively correlated with SDs (r = − .54, p < .01). Length of hospital stay was negatively correlated with QOL (r = − .59, p < .01) and hope (r = − .36, p < .01) but positively correlated with SDs (r = .76, p < .001). Hope was negatively correlated with SDs (r = − .48, p < .01) (Table 3). Age was negatively correlated with QOL and SDs and positively correlated with hope, but the correlations were not statistically significant. (Table 3).

**Table 3:** Correlation among SDs, hope and QOL

	QOL		Hope		SDs	
Variables	r	P	r	p	R	p
Hope	0.78	< 0.001				
SDs	− 0.54	< 0.001	− 0.48	< 0.001		
Age	− 0.04	0.693	0.03	0.769	− 0.18	0.086
Duration (months)	0.20	0.051	0.18	0.079	− 0.09	0.375
Number of TACE treatments	0.14	0.189	0.10	0.333	0.01	0.983
Length of hospital stay (days)	− 0.59	< 0.001	− 0.39	< 0.001	0.76	< 0.001

Note: SDs = Symptom distress, TACE = Trans-hepatic arterial chemoembolization; QOL = quality of life

### Mediation model

According to the results of the bivariate analysis, employment, BCLC stage, and length of stay were significantly associated with QOL; thus, these variables were included in the model as control variables, except the length of hospital stay. The reason for not including the length of hospital stay is that it was highly correlated with the main predictor, SDs, so multicollinearity was a concern. Categorical variables were dummy coded before entering them into the model.

**Fig. 1:**
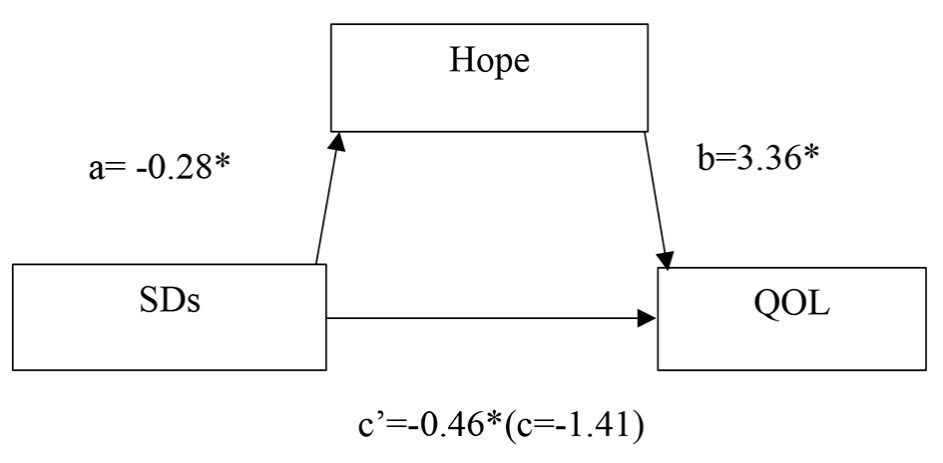
Results of the mediation model. QOL = quality of life, SDs = symptom distress, a = direct effect of SDs on hope, b = direct effect of hope on QOL, c = total effect of SDs on QOL, c’=direct effect of SDs on QOL. * p < .05

The results of the mediation model are presented in Tables 4 and Fig. 1. The total effect of SDs on QOL (c) was significant (B = − 1.41; 95% CI: − 1.96, − 0.86; p < .001). The direct effect of SDs on QOL (c’) was significant (B = − 0.46; 95% CI: − 0.91, − 0.02; p = .041). The direct effect of hope on QOL (b) was significant (B = 3.36; 95% CI: 2.63, 4.09; p < .001). The indirect effect of SDs on QOL mediated by hope (a*b) was significant (B = − 0.95[− 0.28 × 3.36]; 95% CI [bootstrap]: − 1.47, − 0.45, did not include zero). These results indicated hope was a partial mediator.

**Table 4:** Mediation analysis of hope on SDs and QOL

Paths	a	b	c’	a*b	95% CI of a*b	c	SE	R^2^
SDs→hope→QOL	− 0.28	3.36	− 0.46	− 0.95	(− 1.47, − 0.45)	− 1.41	0.28	0.66

Note: SDs = symptom distress, SE = standard error, QOL = quality of life, a = direct effect of symptom distress on hope, b = direct effect of hope on QOL, a*b = indirect effect, c = total effect of SDs on QOL, c’=direct effect of SDs on QOL.

## Discussion

The mean SDs score at discharge of patients receiving TACE was mild in this study. SDs can impair QOL directly and reduce it indirectly by decreasing hope. Hope was a partial mediator.

Participants had moderate levels of overall QOL, which was similar to previous studies [8, 13, 14, 17]. In our study, most patients had acceptable liver function (most of them were in the Child-Pugh A level), but many were unemployed and without income during TACE treatment, which may result in fair social and functional aspects of QOL. Participants had a moderate level of hope, which coincided with prior results [19]. TACE therapy can be performed many times based on a patient’s condition [11]. Receiving TACE on a regular schedule may offer patients hope for the possibility of a cure. The severity of SDs after TACE in this study was similar to that reported in previous studies [13, 17]. TACE can cause severe pain and compromise liver metabolic function [32]; therefore, providing symptom management instruction before discharge is necessary. Moreover, SDs should also be assessed using proper instruments during each admission for treatment.

SDs impairs the QOL of TACE patients directly, which has also been noted before [13, 17]. Additionally, our study revealed that SDs impaired QOL by hindering patients’ hope. Hope functions as a coping mechanism that motivates people to take suitable action to deal with physical and mental problems and prioritize more concrete and realistic goals, which results in positive QOL [7, 18, 21, 22]. However, SDs waned the effect of hope on QOL. Our results coincide with a previous study of 194 patients with different types of cancer; it found that hope could mediate the relationship between psychological stress and health status and the relationship between psychological stress and life satisfaction [33]. Promoting hope among patients with cancer should be a main goal of clinical care. Patients’ hope could be increased through cognitive, affective, and behavioral techniques as well as promoting active control over one’s situation [34]. Hope also could be enhanced by having good communication with healthcare professionals [23]. Symptom management plus hope enhancing strategies are important in care of patients after TACE because ineffective symptom management may lead to hopelessness and decreased QOL, prompting patients to withdraw from treatment.

## Conclusion

SDs can impair QOL directly and reduce it indirectly by decreasing hope. This study addressed the importance of continuity of care in symptom management after TACE. In addition to symptom management, interventions to improve patients’ levels of hope should also be included in the care of patients undergoing TACE. Our cross-sectional, correlational design hinders our ability to infer causality in these relationships. Further, the sample size was relatively small; however, the participants were recruited during their hospitalizations and the post hoc power analysis indicated reliable results. We suggest that future studies employ a longitudinal design and recruit a larger sample.

## Data Availability

The dataset used and analyzed in the current study is available from the corresponding author on reasonable request.

## Abbreviations

ANOVA: Analysis of variance
BCLC: Barcelona Clinic Liver Cancer
CI: Confidence interval
FACT-G: Functional Assessment of Cancer Therapy General
HHI: Herth Hope Index
QOL: Quality of life
SD: Standard deviation
SDs: Symptom distress
SDS-CMF: Symptom Distress Scale-Chinese Modified Form
SE: Standard error
TACE: Trans-hepatic arterial chemoembolization
